# Chlorfenapyr-related delayed rhabdomyolysis: a case series

**DOI:** 10.3389/fneur.2024.1464003

**Published:** 2025-01-08

**Authors:** Lina Xu, Qian Zhou, Yan Li, Sisi Ren, Yubin Hu, Jieru Wang

**Affiliations:** ^1^Emergency Department, Beichen Hospital, Tianjin, China; ^2^Department of Critical Care Medicine, The Fifth People's Hospital of Jinan City, Jinan, China; ^3^Qingyun County People’s Hospital, Qingyun, China

**Keywords:** chlorfenapyr, delayed damage, rhabdomyolysis, acute kidney injury, brainstem injury

## Abstract

**Introduction:**

Chlorfenapyr, a broad-spectrum insecticide and acaricide of the pyrrole-class pesticides, can induce dizziness, fatigue, profuse sweating, and altered consciousness by interfering with cell energy metabolism. However, chlorfenapyr-related rhabdomyolysis has rarely been reported.

**Case presentations:**

Patient 1 was a healthy 26-year-old man who ingested approximately 30 mL of chlorfenapyr. After gastric lavage, rehydration, diuresis, liver protection, and symptomatic treatment, he was discharged. However, he was readmitted 11 days later with rhabdomyolysis and acute kidney injury, and his blood tralopyril level was 187 μg/mL. Patient 2 was a 43-year-old man who consumed approximately 50 mL of chlorfenapyr without seeking medical care for 6 days. On day 7, his blood chlorfenapyr and tralopyril levels were 42 μg/mL and 542 μg/mL, respectively. Subsequently, the patient was diagnosed with rhabdomyolysis and brainstem injury.

**Discussion:**

Chlorfenapyr can disrupt cellular energy metabolism, leading to rhabdomyolysis and brainstem injury, and physical activity may trigger and accelerate rhabdomyolysis. The delayed damage caused by chlorfenapyr poisoning may be attributed to the gradual depletion of cellular energy and prolonged presence of its metabolites in the body.

## 1 Introduction

Chlorfenapyr is a pyrrole pro-insecticide currently used to control insects and mites on various crops (1). As chlorfenapyr does not confer cross-resistance to neurotoxic insecticides (2), it has been gradually popularised in China (3). The toxic form requires oxidation into free pyrrole (4), which uncouples oxidative phosphorylation in the mitochondria, resulting in the disruption of adenosine triphosphate (ATP) production, cellular death, organ damage, and even mortality (5). The central nervous, muscular, and cardiovascular systems, which have high energy demands, are particularly vulnerable to damage (6). Previous reports indicate that chlorphenapyr-related rhabdomyolysis usually occurs after ATP depletion, often accompanied by fever, excessive sweating, and general fatigue (6, 7). To our knowledge, exercise-induced rhabdomyolysis in patients with chlorphenapyr poisoning is rarely reported. Here, we report two cases of chlorfenapyr poisoning patients who developed delayed rhabdomyolysis after some intense physical activity, and a severe case also developed brainstem injury.

This study was approved by the Ethics Committee of Beichen Hospital, Tianjin City. Both patients provided written informed consent to participate in the study, which included a data availability statement.

## 2 Case presentation

### 2.1 Case 1

A previously healthy 26-year-old man ingested approximately 30 mL of chlorfenapyr. Two hours later, he developed nausea and emesis and was transferred to a local hospital for gastric lavage. After 2 days of rehydration, diuresis, liver protection, and symptomatic treatment, the patient was discharged. Five days after discharge, the patient experienced no discomfort. He worked on a construction site for 2 days, during which the intensity of his physical activity was comparable to his previous levels. He gradually developed symptoms such as fatigue, oliguria, dark urine, heat intolerance, and excessive sweating. The patient rested for 1 day, but his condition gradually worsened. He was admitted to our hospital 11 days after the initial ingestion. He had no history of any illness in recent years, including rhabdomyolysis. He had not taken any medications or poisons since his discharge. On admission, the patient was conscious. Despite drinking and eating more than usual, he had lost approximately 2.75 kg in the past 10 days. Physical examination results were normal except for moist skin. His blood tralopyril concentration was 187 μg/mL and blood chlorfenapyr was negative. The major abnormal laboratory results were as follows: white blood cell, 10.5 × 10^9^/L; alanine aminotransferase (ALT), 336 IU/L (range, 0–35); aspartate aminotransferase (AST), 236 IU/L (range, 14–36); blood urea nitrogen (BUN), 15.6 mmol/L; creatinine, 268 μmol/L; creatine kinase (CK) 28,557 ng/mL; and myohemoglobin, 3,029 ng/mL (range, 0–70). Fortunately, the electrocardiogram and brain and chest computed tomography (CT) were normal. The main treatments included continuous venovenous haemofiltration (CVVH), dexamethasone (10 mg, intravenous drip, once a day), pantoprazole (40 mg, intravenous drip, twice a day), reductive glutathione (1.8 grams, intravenous drip, once a day), and hydration therapy. Seventeen days after ingestion, his urine output returned to normal. However, the patient continued to experience heat intolerance and excessive sweating. Subsequent laboratory tests revealed improvements in ALT (96 IU/L), AST (72 IU/L), BUN (8.6 mmol/L), creatinine (98 μmol/L), myohemoglobin (213 ng/mL), and CK (655 ng/mL). The CVVH therapy was discontinued. Twenty-five days after ingestion, the patient was discharged with relief from heat intolerance and sweating. During a follow-up visit 42 days after ingestion, the patient showed no obvious discomfort, and the major laboratory tests were normal.

### 2.2 Case 2

A 43-year-old man ingested approximately 50 mL of chlorfenapyr during a suicide attempt. The patient did not seek medical treatment after ingestion. The next day, the patient drank liquor discontinuously. On days 3 and 4, the patients experienced discomfort. On day 5, he went to work at the removal company, where the workload was similar to that in his previous jobs. However, he felt more fatigued and was likely to sweat and thirst more than usual. After work, the patient felt exhausted and experienced muscle soreness. Subsequently, the patient rested for a day, but his fatigue and sweating were exacerbated, and he developed palpitations. During this period, he had taken 1 table (0.3 gram) of ibuprofen prolonged-release capsules. The patient was transferred to our hospital on day 7. Eight months ago, he had muscle pain and weakness when he began working at a moving company, with CK levels below twice the normal range. He had not taken any poisons or other medications after his discharge. On admission, apart from whole-body skin moisture, apparent facial sweating, and tachycardia (heart rate, 112 beats/min), the patient’s vital signs and physical examination findings were unremarkable. His blood chlorfenapyr and tralopyril levels were 42 μg/mL and 542 μg/mL, respectively. Laboratory tests revealed that the ALT (89 IU/L), AST (129 IU/L), creatinine (138 μmol/L), BUN (13.7 mmol/L), CK (6,491 IU/L), and MYO (1,087 ng/mL) levels were elevated. Results of routine blood examination, procalcitonin level, renal function, thyroid function, electrocardiography, and cardiac ultrasonography were normal. Hemoperfusion and CVVH were performed. His medical treatment was similar to that of patient 1. On day 8, his weakness continued to worsen, and he developed drowsiness. He developed a low-grade fever in the morning, which progressed to high fever at night followed by ibuprofen and carpet. On day 9, the patient continued to experience high fever and profuse sweating. The laboratory test results were as follows: ALT, 192 IU/L; AST, 77 IU/L; myoglobin, 762 ng/mL; CK, 2575 ng/mL; and CK-MB, 152 ng/mL (range, < 4). Electrocardiography revealed tachycardia (rate, 124 beats/min) and broad ST-segment depression. His chest CT was normal, but brain CT showed low density in the pons, bilateral brachium pontis, and pedunculus cerebri. On day 10, the patient developed apparent tachypnoea and delirium, and endotracheal intubation and mechanical ventilation were performed. Eleven days later, he developed hypotension and oliguria, norepinephrine was applied. In the afternoon, he developed generalised convulsions. Diazepam and a muscle relaxant (vecuronium bromide) were administered, and the convulsions were controlled 10 min later. Repeated laboratory tests were as follows: creatinine, 176 μmol/L; BUN, 16.9 mmol/L; ALT, 231 IU/L; AST, 177 IU/L; myoglobin, 1728 ng/mL; and CK, 11,562 ng/mL. Unfortunately, ventricular fibrillation occurred 2 h later, and spontaneous circulation was restored after defibrillation. Cardiac arrest occurred 35 min later, and the patient died after positive rescue.

## 3 Discussion

The common clinical features in both patients were fatigue, excessive sweating, and rhabdomyolysis. Additionally, acute kidney injury, weight loss, fever, myocardial damage, and toxic encephalopathy were the clinical findings of this study. Chlorfenapyr poisoning has been reported to have an initial latent period lasting 7–14 days after ingestion (8). The two patients separately presented with obvious clinical symptoms on day 8 and day 5 after ingestion, despite their intake amounts of only 30 mL and 50 mL, respectively. In this paper, the tralopyril levels of case 1 and case 2 were 187 and 542 μg/mL on days 11 and 6, respectively. Tralopyril is the main toxic metabolite of chlorfenapy and can persist in the body. Its concentration is related to clinical manifestations (9, 10), consistent with our observations.

Rhabdomyolysis is a life-threatening condition in which the skeletal muscle tissue breaks down, releasing muscle cell contents such as myoglobin and CK into the blood circulation (11), which can include muscle pain, weakness, swelling, and dark or red-coloured urine. Convulsions, unconsciousness, and compression are common causes of rhabdomyolysis (11, 12). Both patients did not have a fever and exhibited excessive sweating before the physical activity and rhabdomyolysis, suggesting that ATP depletion was not complete (7). After treatment, case 1 reported a rapid decrease in CK and myoglobin levels, while Case2 initially showed a slight reduction followed by an increase. The persistently elevated CK and myoglobin levels in case 2 may be associated with ATP depletion. Physical activity can excessively consume energy; therefore, we speculate that it may shorten the latent period, and trigger and exacerbate rhabdomyolysis and organ injuries. Weight loss has been previously mentioned in the literature, and we identified its presence in case 1 (9). This may be associated with excessive fluid loss and uncoupling, leading to excessive energy expenditure and downregulation of protein biosynthesis in the body (13). The acute kidney injury may be related to hypovolaemia caused by sweating and rhabdomyolysis. Brainstem injury is frequently mentioned in cases of chlorfenapyr poisoning (8, 14), and we observed abnormal changes in the pons, bilateral brachium pontis, and pedunculus cerebri in case 2. Case 2 also developed myocardial injury with elevated myocardial enzyme levels and broad ST-segment depression on electrocardiography. Brainstem and myocardial injuries may be related to the high-energy demands of these tissues and regions, making them more susceptible to energy deprivation and damage.

There are no specific antidotes for chlorfenapyr poisoning. Early positive gastric lavage, adsorption, catharsis, hemoperfusion, and symptomatic supportive treatment may be efficient treatments (15, 16). This could reduce blood chlorfenapyr levels and further reduce the conversion of chlorfenapyr to tralopyril. Fever and excessive sweating are the late-stage toxicities of chlorfenapyr poisoning, which indicated that the patient had already lacked energy, suggesting a serious condition and an increased risk of death (7). Chomin et al. (17) suggested that supportive care, antioxidant therapy, and late haemodialysis may be futile once late toxicity is established. Owing to the initial latent period, early intervention may be necessary, even in patients without obvious clinical symptoms after ingestion. Tralopyril can persist in the body, leading to clinical symptoms and injury. Hence, removing tralopyril through haemopurification treatments long after exposure might still effectively improve symptoms and prognosis.

## Data Availability

The datasets presented in this article are not readily available because of ethical and privacy restrictions. Requests to access the datasets should be directed to the corresponding author.
